# The Effect of Preoperative Educational Intervention on Anxiety and Pain of Patients Undergoing Spinal Decompression Surgery: A Pilot Randomized Controlled Study

**DOI:** 10.7759/cureus.28368

**Published:** 2022-08-25

**Authors:** Veronika Feninets, Theodoula Adamakidou, Marianna Mantzorou, Dimos Mastrogiannis, Ourania Govina, Chrysoula Tsiou

**Affiliations:** 1 Nursing Department, University of West Attica, General Hospital of Nikaia “Agios Panteleimon”, Athens, GRC; 2 Nursing Department, University of West Attica, Athens, GRC; 3 General Department, University of Thessaly, Athens, GRC

**Keywords:** anxiety, pain, spinal surgery, nursing educational intervention, preoperative education, patient education

## Abstract

Background: Preoperative patient education is an effective intervention of the healthcare team, which has been used to promote patient recovery and well-being.

Aim: The aim of this study was to investigate the effect of a nursing preoperative educational intervention on the anxiety and pain of patients undergoing spinal decompression surgery.

Methods: In this pilot randomized controlled study, patients (n=40) undergoing spinal decompression surgery, were randomized into an intervention group (underwent educational intervention, n=23) and a control group (n=17). The preoperative educational intervention included an oral briefing and a leaflet with perioperative care information as well as post-discharge care. Participants completed the Amsterdam Preoperative Anxiety and Information Scale (APAIS), the subscale “State” of the State and Trait Anxiety Inventory (STAI-S), the Numerical Rating Scale, and a questionnaire about demographic characteristics, the day before surgery (T0) and the first post-operation day (T1). Data analysis was conducted using SPSS 22.0. The statistical significance level was set at p<0.05.

Results. A significant reduction was found in pain intensity before and after surgery in both groups. Pain levels, after surgery, were significantly lower in the intervention group compared to pain levels in the control group (t=2.174, p=0.036). In both groups, high state anxiety scores on the STAI-S scale before surgery were confirmed by high anxiety scores in APAIS_surgery_. Additionally, in both groups after surgery, high state anxiety scores on the STAI-S scale were associated with high pain levels. There were no statistically significant group differences with regard to scores of STAI-S before and after surgery.

Conclusions. Nursing preoperative educational intervention in patients undergoing spinal decompression surgery had a positive impact on reducing the intensity of pain after surgery. These results indicated that nurses and health care providers should integrate patient education and health literacy into their daily clinical practice.

## Introduction

Spinal decompression surgery is commonly offered to patients with intervertebral disc herniation and lumbar stenosis when conservative management of symptoms is not effective. These pathological conditions have a high prevalence [1] and negatively affect patients' quality of life due to the intense pain they cause [2]. Factors such as pain, information, disability, employment, and mental health seem to be of great concern to patients undergoing spine surgery and are associated with high levels of anxiety and depression. Moreover, the role of patients’ knowledge and information has been pointed out as a regulating factor for anxiety and depression coming from the above patients’ concerns [3].

Nurses often use educational interventions to reduce patients’ anxiety [4,5], focusing on supporting self-management, encouraging self-care, enhancing active participation in decision-making, providing emotional support and skills training to improve health and well-being, and generally promoting patients’ active role in their treatment [6-8]. Preoperative educational interventions in patients undergoing spine surgery have been found effective in reducing postoperative anxiety [5,7,9], pain [5], and the use of analgesia [10]. However, it has been argued that studies supporting the beneficial clinical, economic, and psychological outcomes of preoperative surgery educational interventions, although of “fair” methodological quality, are limited, with small sample sizes and diversity both in surgical procedures as well as in educational interventions [11].

During the pandemic, time constraints and severe nursing staff restrictions in Greece resulted in limited preoperative educational briefings between patients and healthcare professionals which were generally limited to the surgical procedure itself. Thus, patients who received standard or usual care were informed by the surgeon and signed the consent form. The consent form is a legal requirement before surgery, conducted exclusively by the surgeon, emphasizes information about the surgical procedure, the outcomes, and the potential complications, and ensures patients’ consent for the surgery [12]. On the other hand, preoperative educational intervention is an interactive procedure, that focuses on the improvement of patient self-care and self-efficacy after the surgery and is usually conducted by a nurse [13]. Protocols regarding preoperative education are available in Greek surgical units, but empirical data show that there is a variation in their individualized and systematic implementation among clinics and hospitals.

The aim of this study was to investigate the effect of a systematic preoperative educational intervention on the levels of stress and pain experienced by patients who underwent spinal decompression surgery.

## Materials and methods

Study design

This pilot study was a randomized controlled pilot study, which includes an intervention group (IG) and a control group (CG) with pre-intervention and post-intervention measurements.

Sample and procedure

The sample of this pilot study consisted of 40 patients in total, suffering from intervertebral disc herniation or lumbar stenosis. Patients were admitted to the neurosurgery clinic in a tertiary hospital in Athens, Greece, to undergo microdiscectomy or laminectomy during the time period from November 2020 to October 2021. Patients were randomly allocated to either the CG (17 patients) or the IG (23 patients) based on the admission date and received an individualized educational intervention. Patients admitted on an odd date were allocated to the CG, while patients admitted on an even date were allocated to the IG. None of the participants knew which group they belonged to. The inclusion criteria were: age > 18 years, impending spinal decompression surgery (microdiscectomy or laminectomy), ability to speak and understand the Greek language, and provision of consent to participate in the study.

**Figure 1:**
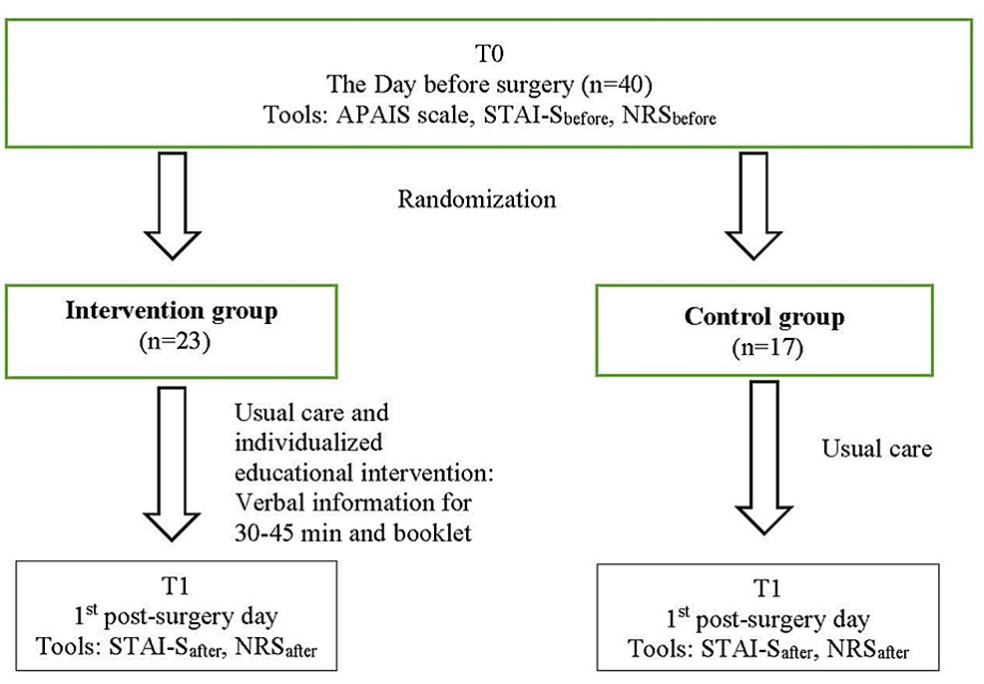
Study's flowchart

Prior to the surgery, participants were informed about the study and signed the consent form for their participation. One day before surgery (T0) all participants completed the questionnaire (Figure 1).

Patients of the CG after completion of the questionnaire (T0) received standard care from the healthcare providers and did not receive educational intervention from the researcher. In the afternoon of the first postoperative day (T1) (24-30 hours after the surgery) they completed again the same questionnaire, except for the Amsterdam Preoperative Anxiety and Information (APAIS) since it measures patients' anxiety and needs for information in the preoperative period.

Patients in the IG, immediately after the completion of the questionnaire (T0), received the educational intervention which included oral conversation and provision of information lasting about 30-45 minutes concerning preoperative, intraoperative, and postoperative nursing care. A leaflet containing the same information was given to patients as well. In the afternoon of the first postoperative day (T1), patients completed the same questionnaire again, except for the APAIS scale.

Educational intervention

Preoperative education is a nursing intervention that clinical nurses and other team members apply in order to enhance patient self-efficacy. The design and preparation of the current preoperative educational intervention were based on the current trends (early scheduling of the intervention, messages to be repeated (oral briefing and leaflet), the content of the intervention included post-operation management and appropriate follow-up) [13].

An oral briefing was made by the primary researcher, who is a nurse with long clinical experience in the neurosurgery clinic and the nursing care of these patients. The educational intervention was based on the leaflet which was created for the purposes of this study and was given to patients. The leaflet and therefore the oral briefing consisted of four sections aiming to increase patients’ health literacy. The first section consisted of information about intervertebral disc herniation and lumbar stenosis. The second concerned the patient’s preoperative preparation (preoperative procedures, venipuncture, pre-anesthesia feeding, incision site, stitches and removal time, etc.). The third section included informing the patient about the care and procedures in the intraoperative phase (identification, duration of surgery and type of anesthesia, etc.). Finally, the fourth section concerned the patient’s postoperative care during hospitalization as well care following hospital discharge (medication and analgesic interventions, permitted range of mobilization in the first 24 hours and thereafter, precautions during mobilization, activities of daily living, wound care information, use of an orthopedic belt, various points needing attention at home and ergonomic guidelines during driving, sitting, exercise, sexual life, etc.).

The leaflet was designed by the primary researcher and finalized with the contribution of the entire research team, based on their long clinical experience and the available literature. During the preparation of the educational material, the researcher took into account questions, queries, and remarks related to the specific surgery that are formulated by the patients who are being treated in the unit where she works. The first version of the material was then given for evaluation to an academic with knowledge of providing nursing care for patients undergoing spine surgery, to a clinical nurse with many years (> 15 years) of experience in the nursing care of patients undergoing spine surgery, and to 2 patients who were not included in the present sample. Then, following the comments, corrections, and modifications acknowledged by the specialists and the patients, the educational material took its final form and was printed out. The printed educational material contains images, is comprehensible to readers, and is written in a language that is simple and easy for them to understand.

Measures

The questionnaires used in the present study were the following. A questionnaire regarding demographic data was constructed for the study (gender, age, level of education, marital status, employment status, type of operation, history of previous surgery under general anesthesia, and the main caregiver).

The State and Trait Anxiety Inventory (STAI-S) scale measures state (adaptive response to a situation) and trait anxiety (reflecting personality traits) [14]. Only the state anxiety scale was used since the emphasis of our study was to examine anxiety caused by the surgery. It consists of 20 questions. Each item is answered on a 4-point Likert-type scale (1=almost never to 4=almost always). The scale’s range of values varies from 20 (lowest anxiety) to 80 (the highest anxiety). The Greek version was validated by Fountoulakis et al. and the Cronbach’s alpha correlation coefficient was 0.93 [15].

The APAIS measures patients' anxiety and need for information during the preoperative period [16]. The 6 items were answered in a five-point Likert-type response format (1=not at all, 5=too much). The scale includes two subscales, APAIS anesthesia which measures anxiety and needs for information concerning the anesthesia, and APAIS surgery which measures anxiety and need for information related to surgery. The Greek version was validated by Bakalaki et al. and Cronbach’s alpha correlation coefficient was >0.8 for both subscales [17].

The Numeric Rating Scale (NRS) was used to assess pain. A value of 0 represents “no pain at all” and a value of 10 represents “the worst pain there can be.” Values ranging from 1 to 3 reflect mild pain, 4 to 6 reflect moderate pain and 7 to 10 reflect severe pain [18].

Ethical considerations

This study was approved by the Scientific Committee of the Nikaia’s Hospital in Athens, Greece (Ref No: 53563/25-11-2020). Written informed consent was obtained by all participants. Helsinki declaration principles were abided by in this study. Permissions were obtained for the use of STAI and APAIS scales by their developers.

Statistical analysis

Statistical analysis of the study’s variables was performed using Statistical Package for Social Sciences (SPSS) version 22.0 (IBM Corp., Armonk, NY, USA). Shapiro-Wilk's test was employed to examine whether quantitative variables followed a normal distribution, due to our small sample size. T-test was used to examine the presence or absence of relationships between a quantitative variable and a dichotomous variable. Pearson’s correlation coefficients were used to examine the relationship between two quantitative variables. In order to examine the questionnaires’ reliability, Cronbach’s α value was calculated. Cronbach’s α value for the STAI questionnaire was 0.938 and for the APAIS questionnaire was 0.771. These values are considered satisfactory (APAIS) to very good (STAI). The level of statistical significance was set to p<0.05.

## Results

Our sample consisted of 40 patients, 23 of them, 13 men and 10 women, were allocated to the IG. The rest 17 (seven men and 10 women) were allocated to the CG. There were no significant differences in demographic and clinical characteristics between the two groups (Table 1). Also, there were no significant differences between the two groups at baseline levels of anxiety and pain prior to surgery (Table 2). In the IG, reduction of pain mean values were greater compared to the CG after surgery.

**Table 1:** Patient demographic characteristics.

Demographic characteristic	Control group		Intervention group	
	Ν	%	Ν	%
Gender				
Male	7	41.2	13	56.5
Female	10	58.8	10	43.5
Age (Μean±SD)	48.47±13.8		52.39±13.5	
Marital Status				
Unmarried-Divorced-Widow/er	8	47.1	9	39.1
Married	9	52.9	14	60.9
Educational Level				
Mandatory education (12 years)	11	64.7	16	69.6
Tertiary education	6	35.3	7	30.4
Profession				
Employed	10	58.8	14	60.9
Unemployed	2	11.8	2	8.7
Retired	2	11.8	5	21.7
Housework	3	17.6	2	8.7
Type of surgery				
Microdiscectomy	15	88.2	19	82.6
Laminectomy	2	11.8	4	17.4
History of previous surgeries under general anesthesia				
Υes	15	88.2	14	60.9
No	2	11.8	9	39.1

 

**Table 2:** Levels of pain, state anxiety, and preoperative anxiety on T0 and T1 for the intervention and control group.

	Before						After				
Characteristic	Control group			Intervention group			Control group		Intervention group		
	Ν	%		Ν		%	Ν	%	Ν	%	
Numeric rating scale											
No pain (0)	0	0		1		4.3	0	0	2	8.7	
Mild pain (1 to 3)	3	17.6		5		21.7	9	52.9	14	60.9	
Moderate pain (4 to 6)	9	52.9		12		52.2	6	35.3	7	30.4	
Severe pain (7 to 10)	5	29.4		5		21.7	2	11.8	0	0	
Numeric rating scale (mean±SD)	6.24 ± 2.86			5.74± 2.49			4.59±0.71		2.22±0.60		
STAI-S (mean±SD)	1.94±0.65			1.96±0.76			2.12±0.69		1.70±0.63		
Mild	10		58.8	11	47.8		10	58.8	17		73.9
Moderate	7		41.2	10	43.5		7	41.2	6		26.1
High	0		0	2	8.7		0	0	0		0
APAIS scale											
APAIS_anesthesia_	6.76±4.101			6.96±2.755			-		-		
APAIS_surgery_	9.53±2.939			8.91±3.541			-		-		

After performing Kendall’s tau test (Table 3), it was found that before surgery in both groups, high state anxiety scores on the STAI-S scale were confirmed by high anxiety scores in APAIS_surgery_ (p=0.009 and p=0.018 for CG and IG, respectively). Additionally, after surgery, high scores on the STAI-S scale were associated with high pain levels as demonstrated by NRS scale values, in both groups (p=0.001 and p=0.02 for CG and IG, respectively).

**Table 3:** Association between STAI-S preoperatively and postoperatively with APAISsurgery and NRSafter in both groups. τ*: Kendall’s tau

	STAI-S_before_					STAI-S_after_				
	Control group			Intervention group		Control group			Intervention group	
	τ*	P-value		τ*	P-value	τ*	P-value		τ*	P-value
APAIS_surgery_	0.485		0.009	0.368	0.018	-		>0.05	-	>0.05
NRS_after_	-		>0.05	-	>0.05	0.649		0.001	0.371	0.02

Using the t-test procedure, a significant reduction was found in pain intensity (NRS scores) before and after surgery in both groups (Table 4). High pain intensity before surgery was correlated with high pain intensity after surgery in both groups.

**Table 4:** Comparison between NRS of Control group and Intervention group before and after surgery. *dependent t-test

	NRS_after_			
	Control group		Intervention group	
	t*	p-value	t*	p-value
NRS_before_	3.926	0.001	3.579	0.002

The comparison of the scores of scales between the two groups before and after surgery (Table 5), showed that there was a significant difference in the intensity of pain scores (NRS) after surgery (t=2.174, p=0.036) in the IG. This means that since pain levels did not differ between the two groups before surgery, the intensity of pain was reduced more in the IG compared to the CG after the surgery. There were no statistically significant group differences with regard to scores of STAI-S before and after surgery, pain intensity before surgery, as well as the two APAIS subscales (APAIS_anesthesia_ and APAIS_surgery_) (Table 5).

**Table 5:** Comparison between state anxiety score, pain and perioperative anxiety in control and intervention groups. *Mann-Whitney U Test

	t-test	p-value
STAI-S_before_	-0.380	0.706
STAI-S_after_	1.068	0.292
NRS_before_	0.585	0.562
NRS_after_	1.759	0.037
APAIS_anesthesia_	225.500*	0.407
APAIS_surgery_	0.584	0.563

## Discussion

This study investigated the effect of preoperative educational intervention on the stress and pain experienced by patients undergoing spinal decompression surgery and demonstrated that educating patients preoperatively can reduce the pain they experience postoperatively.

The most important finding of this pilot study was that preoperative educational intervention significantly reduced postoperative pain in patients who underwent spinal decompression surgery compared to patients who did not receive preoperative education. This finding is consistent with the randomized study of Lee et al. in patients who underwent spine surgery [5] and another study of patients who underwent total knee arthroplasty [19]. Similar findings have been reported by Rahmani et al. who found a reduction in pain intensity and a reduced need for opioid analgesics in the postoperative phase after training intervention in 46 patients who underwent orthopedic surgery [20]. In addition, higher satisfaction with pain management was reported in the IG of patients undergoing elective spinal surgery compared to the untrained CG [10]. Conversely, the absence of a reduction in pain intensity of patients in the postoperative educational group compared with patients in the non-educational group was found in a study on 220 patients who underwent shoulder or breast surgery. Specifically, the psycho-educational intervention was applied on the second, third, and seventh postoperative days and not preoperatively as in the above studies [21]. Therefore, the timely application of educational intervention is an important factor in its effectiveness. For this reason, it is suggested that patient education should be part of the routine of preoperative nursing preparation of patients in order to relieve postoperative pain [22].

In the present study, a reduction in postoperative pain intensity was found in both groups. In the IG, the reduction of pain is probably attributed to the intervention and postoperative analgesia. In the group that received the usual preparation for surgery without education, the reduction in postoperative pain may be related to postoperative analgesia alone. However, the statistically significant difference found in postoperative pain between the two groups strongly indicates the positive effect of preoperative education in the IG group. It would be interesting in future studies to control for the type and amount of postoperative analgesia, as these were not identified in the context of the present pilot study.

The results suggest that in both groups, high postoperative state anxiety was associated with high postoperative pain intensity. The relationship between anxiety and pain is also confirmed by another study where a positive significant correlation between anxiety and pain was found in patients after total knee or hip arthroplasty and they concluded that the most important prognostic factor for postoperative anxiety is preoperative anxiety [23]. A previous study in patients (n=712) who underwent various types of surgery found that moderate to severe postoperative pain as well as preoperative anxiety were risk factors for postoperative anxiety [24]. High anxiety levels appear to correlate with pain, as anxious patients are more sensitive to pain [25].

The present study found no statistically significant group differences in stress reduction (STAI-S), before and after surgery. This finding may be attributed to the small study sample in both groups. Consistent with our findings, several studies do not reveal a significant influence of the educational intervention on the level of anxiety of patients undergoing spine surgery [11], thyroidectomy [26], general surgical patients [27], patients undergoing shoulder and breast surgery [21]. On the other hand, there are studies that have shown a significant influence of educational intervention on the anxiety level of patients undergoing spinal surgery [5,7,9]. Given the mixed findings of the effect of preoperative educational intervention on patients' postoperative anxiety, further studies with homogeneous large samples will contribute to a better investigation and exploration of this relationship.

The current pilot study suggests that increased state anxiety before surgery (STAI-S_before_), in both groups, was associated with increased anxiety for the surgery (APAIS_surgery_); as it is stated in a previous study that the correlation of the APAIS scale with the STAI-S scale is an important indicator of the validity of the APAIS scale [17].

Limitations and strengths of the study

The present research is a pilot study with a small sample size in both the IG and the CG. Data collection was performed during the SARS-CoV-2 pandemic, a time period where only emergency surgeries were conducted, a fact that led to severe difficulty in acquiring a larger sample as well as marked delays in questionnaires’ distribution and collection. A larger and more representative sample may have yielded more insight regarding relationships between variables’ correlations and may have led to more concrete results. Another limitation is the origin of the sample from only one hospital, a fact that limits the sample’s representativeness and generalizability. A multi-center study could have yielded more reliable results thus allowing for their generalization. One more limitation was the fact that data were collected only on the first post-operative day, due to the patients’ very short length of hospital stay. Follow-up measurements could have strengthened our results.

Despite the above limitations, the homogeneous delivery of the intervention which was conducted exclusively by the primary researcher, as well as the fact that the sample of patients underwent similar, in extent and severity, surgical procedures constitute strengths that add to the rigor of our study.

## Conclusions

In conclusion, the results of this pilot randomized intervention study add new evidence of the effectiveness of the preoperative educational intervention as a strategy to reduce the intensity of pain experienced postoperatively by patients undergoing spine decompression surgery. Patient education and health literacy promotion, are an integral part of nursing care and its implementation could be facilitated by the development of education protocols for procedures performed, such as preparation for surgery, or clinical examination. The results of the current as well as similar studies which show the effectiveness of preoperative education may increase awareness of nurses and health care professionals about the importance of integration of documented educational interventions in daily clinical practice.
